# Hydrophobic durability characteristics of butterfly wing surface after freezing cycles towards the design of nature inspired anti-icing surfaces

**DOI:** 10.1371/journal.pone.0188775

**Published:** 2018-01-31

**Authors:** Tingkun Chen, Qian Cong, Yingchun Qi, Jingfu Jin, Kwang-Leong Choy

**Affiliations:** 1 Key Laboratory of Bionic Engineering, Ministry of Education, Jilin University, Changchun, Jilin Province, P.R., China; 2 State Key Laboratory of Automotive Simulation and Control, Jilin University, Changchun, Jilin Province, P.R., China; 3 College of Biological and Agricultural Engineering, Jilin University, Changchun, Jilin Province, P.R., China; 4 Institute for Materials Discovery, University College London, London, United Kingdom; University of Akron, UNITED STATES

## Abstract

The hydrophobicity and anti-icing performance of the surfaces of some artificial hydrophobic coatings degraded after several icing and de-icing cycles. In this paper, the frost formation on the surfaces of butterfly wings from ten different species was observed, and the contact angles were measured after 0 to 6 frosting/defrosting cycles. The results show that no obvious changes in contact angle for the butterfly wing specimens were not obvious during the frosting/defrosting process. Further, the conclusion was inferred that the topography of the butterfly wing surface forms a special space structure which has a larger space inside that can accommodate more frozen droplets; this behavior prevents destruction of the structure. The findings of this study may provide a basis and new concepts for the design of novel industrially important surfaces to inhibit frost/ice growth, such as durable anti-icing coatings, which may decrease or prevent the socio-economic loss.

## Introduction

Frost formation on solid surfaces is ubiquitous in nature, and occurs when water vapour droplets make contact surfaces with having temperaturess lower than the freezing point of water. Such frost formation cannot be avoided and can be detrimental. Indeed, frost formation on the surfaces of airplanes, heat exchangers, refrigeration and cryogenics equipments, for examples, often degrades the operational efficiency of these machines, even impairs the performance and generates safety risks [1, 2].

Various active methods have been developed to solve or reduce freezing adhesion hazards, which include mechanical and thermal methods to remove icing surfaces. In addition, passive methods have also been explored, including surface chemical modification methods. Superhydrophobic surfaces (SHS) are regarded as potential “anti-icing/anti-frosting method” owing to their extraordinary water-repellency and low surface energy [3]. Therefore, one approach to suppression of frost formation is through design of (super) hydrophobic surfaces to inhibit frost/ice growth and optimise applications of existing anti-frosting methods.

The frost formation process is complex because the frost properties vary continuously during the formation process. Hayashi et al. [4] have proposed an initial one-dimensional (1-D) frost crystal growth process in which frost formation is divided into the growth period and full-growth period. In the “early stage” of frost formation, water vapour is deposited on a cold surface forming very small droplets. After the early-stage, as the surface temperature continues to decrease, the droplets are frozen and water vapours adheres to the frozen droplets forming frost. Studies on the early-stage of frost formation are helpful in elucidating the initial conditions required for the subsequent frosting event. In order to comprehensively study the important factors affecting the frost formation process, such as the substrate and ambient temperature, many researchers have proposed the theoretical models to predict the relationship between these factors and frost formation [5–8]. Satisfactory agreements have been achieved between the predictions of these models and test data. In particular, Na and Webb [9, 10] have systematically studied frost nucleation on flat surfaces, and examined the factors affecting the frost formation. Hence, they found that the surface topography has the greatest influence on this behaviour.

As noted above, the creation of hydrophobic surfaces as a potential method of restraining or alleviating frost formation on a cold surface has been explored in literature [11–13]. Previously, Seki et al. [14] studied the early-stage of frost formation on two surfaces with contact angles of 110° and 43°, respectively, and found that the freezing time was significantly delayed on the hydrophobic surface. Further, Tourkine et al. [15] investigated the delayed freezing of water droplets on water repellent materials. Kulinich et al. [16–19] performed a study of the anti-icing performance of some hydrophobic surfaces, and found that the hydrophobicity and anti-icing performance degrades during repeated icing and de-icing cycles. Those findings are in agreement with the experimental results of other studies [20, 21].

The findings of the above studies can be summarised as follows: Hydrophobic surfaces, including surfaces with hydrophobic coatings and materials created through modification of the surface morphology and microstructure, have significant icing and frosting inhibition properties. However, the durability of a hydrophobic surface is very poor and the micro-structure is destroyed after several testing cycles. In contrast, structures appearing in nature are optimised and tailored to the living environment, resulting from thousands of years of evolution. For example, the characteristics of a butterfly wing surface, including hydrophobicity, stealth and fluorescence, have developed to provide strong performance in high-humidity and low-temperature environments.

In this study, the typical hydrophobic surfaces of various butterfly wings are examined, focusing on their ability to sustain repeat frosting/defrosting cycles and to elucidate the durability mechanism. The findings of this analysis provide new design concepts towards improving the durability of superhydrophobic coating or for the fabrication of anti-icing surfaces with excellent durability.

## Materials and methods

### Experimental apparatus

**Fig 1:**
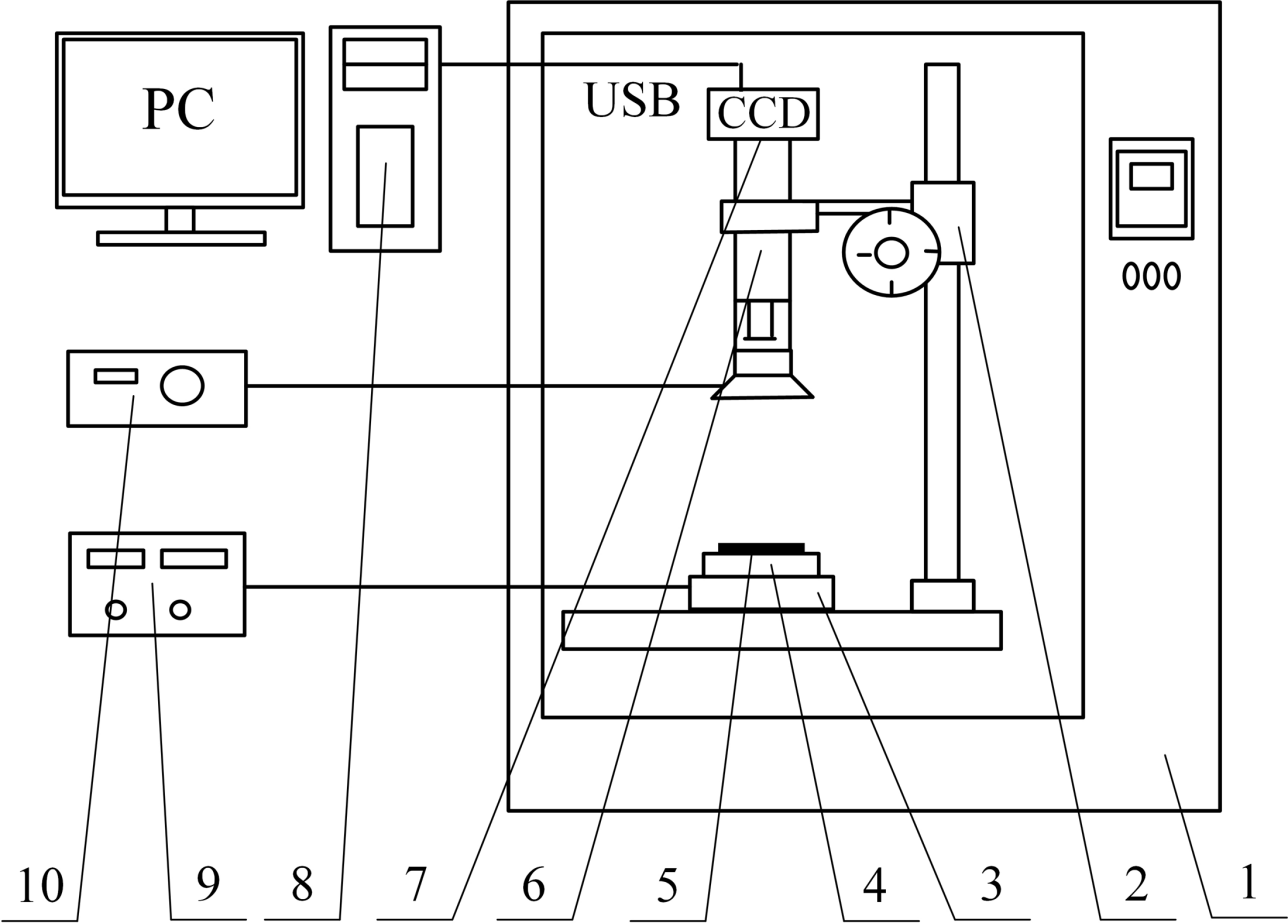
Experimental setup for study of frosting/defrosting cycles on butterfly-wing surface. 1.climate chamber; 2.focusing mechanics; 3.cooling table; 4.copper plate; 5.sample; 6.microscope; 7.CCD camera; 8.PC(acquisition system); 9.temperature controller; 10.lighting system.

A schematic diagram of the experimental setup used to study the frosting/defrosting cycles on the surface of a butterfly wing is shown in Fig 1. The apparatus consisted of a microscope, charge-coupled device (CCD) camera, cooling system and data acquisition system. The cooling system was comprised of a cooling table and low-temperature reaction bath with a temperature range of -30°C to 0°C. The substrate was a 4 cm×4 cm copper plate (Xingye Copper International Group Limited) with 3mm thickness. The surface temperature of the substrate was measured by a K-type thermocouple, which was positioned on the substrate and as close as possible to the sample. The accuracy of the thermocouple was ±1.0%. The frost formation process was recorded by the microscope (with the CCD camera), which was placed above the substrate. The image sequence was recorded in the computer by the data acquisition system, and the time interval between two images was two seconds.

During the experiments, the entire experimental setup was placed in a climate chamber equipped with a temperature and humidity control system. The inner dimensions of the chamber were 60 cm × 50 cm × 100 cm (L × W × H). The experiments were conducted under natural convection conditions, at constant relative humidity and temperature set to 80% and 18°C, respectively.

### Materials

The butterfly species employed in the experiment are not nationally protected or scarce, and the specimens were purchased from Yunnan Aegean Butterfly Cultivation Garden Co., Ltd. The seller completed preliminary distinction and identification of the butterfly specimens.

**Fig 2:**
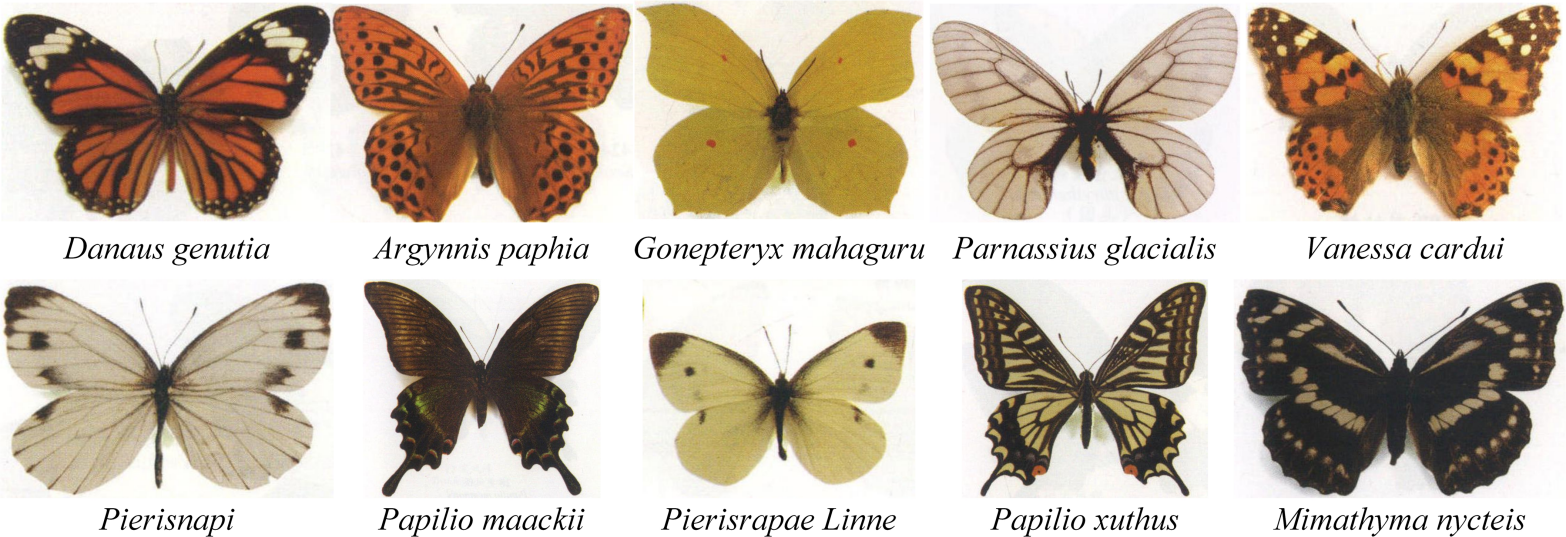
Ten different butterfly species considered in the present study.

Ten butterfly species with hydrophobic wing surfaces were selected for the experiment. With reference to the literature [22–24], the purchased butterfly specimens were identified based on systematic taxonomy to be further distinguished and classified. The butterflies considered in this study were respectively *Papilio maackii*, *Gonepteryx mahaguru*, *Papilio xuthus*, *Vanessa cardui*, *Pieris napi*, *Argynnis paphia*, *PierisrapaeLinne*, *Parnassius glacialis*, *Mimathyma nycteis*, *Danaus genutia*, as shown in Fig 2. The upper surfaces of the butterfly wings were used in the experiments.

**Fig 3:**
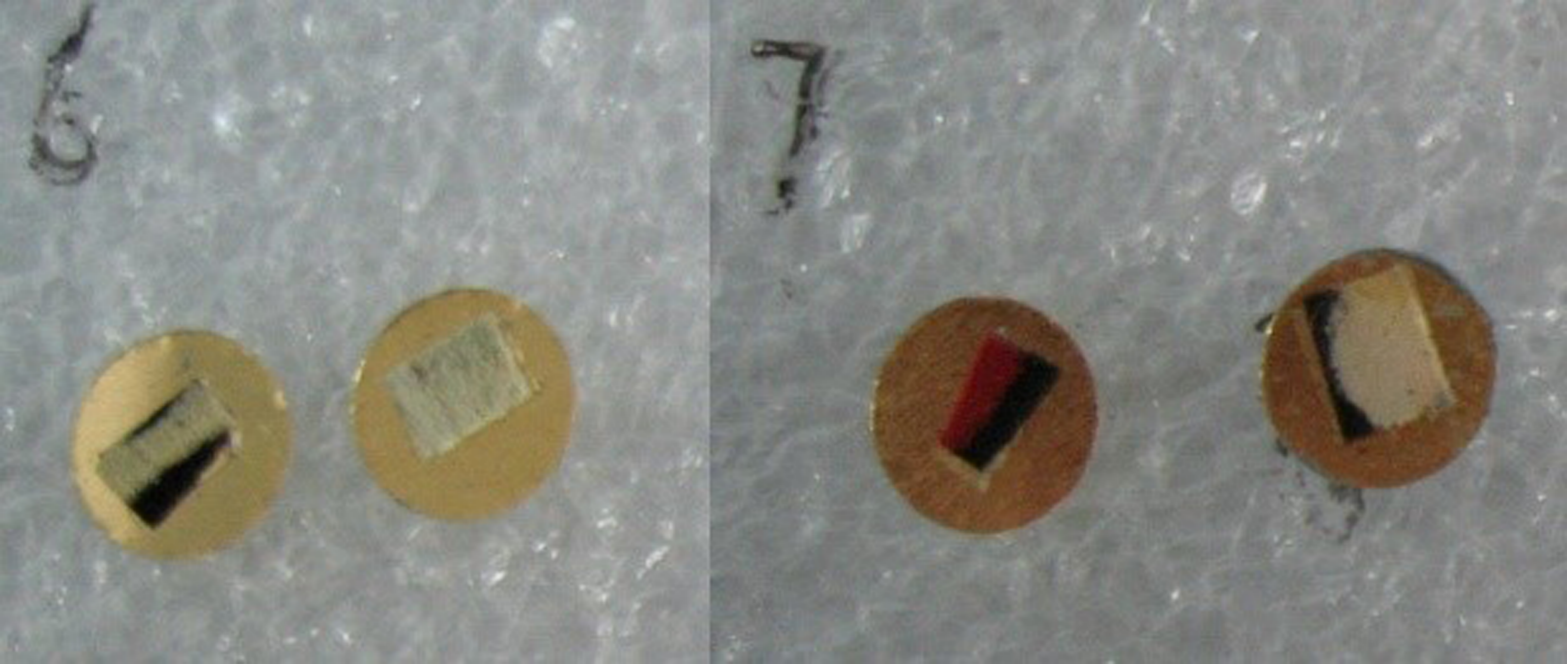
Prepared butterfly wing samples.

Small specimens were cut from the butterfly wings and mounted onto copper sheets using 3M 8810 thermal conductor adhesive tape, as shown in Fig 3. The diameter and thickness of the copper sheets were 6 mm and 0.5 mm, respectively. Before each test, the copper substrate was cleaned with acetone (Beijing Chemical Works), and the butterfly wing sample was adhered to the copper substrate with thermally conductive grease to fill the microscopic air-gap between the two surfaces.

### Experimental procedures

Surface hydrophobicity is commonly quantified by the contact and roll angles; however, the roll angle is often used to characterise the self-cleaning ability of the material. Therefore, in this study, the contact angle alone was used to evaluate the wettability of the butterfly wing. A contact angle measurement system (OCA 30–2, Data-physics Instruments GmbH, Germany) equipped with a tilting base device was employed. The sessile-drop method was also used, through which a 5 *μl* drop of water at ambient temperature (18°C) and 60% humidity was applied to the butterfly wing surface. The water contact angles were measured after every frost/defrost cycle.

**Fig 4:**
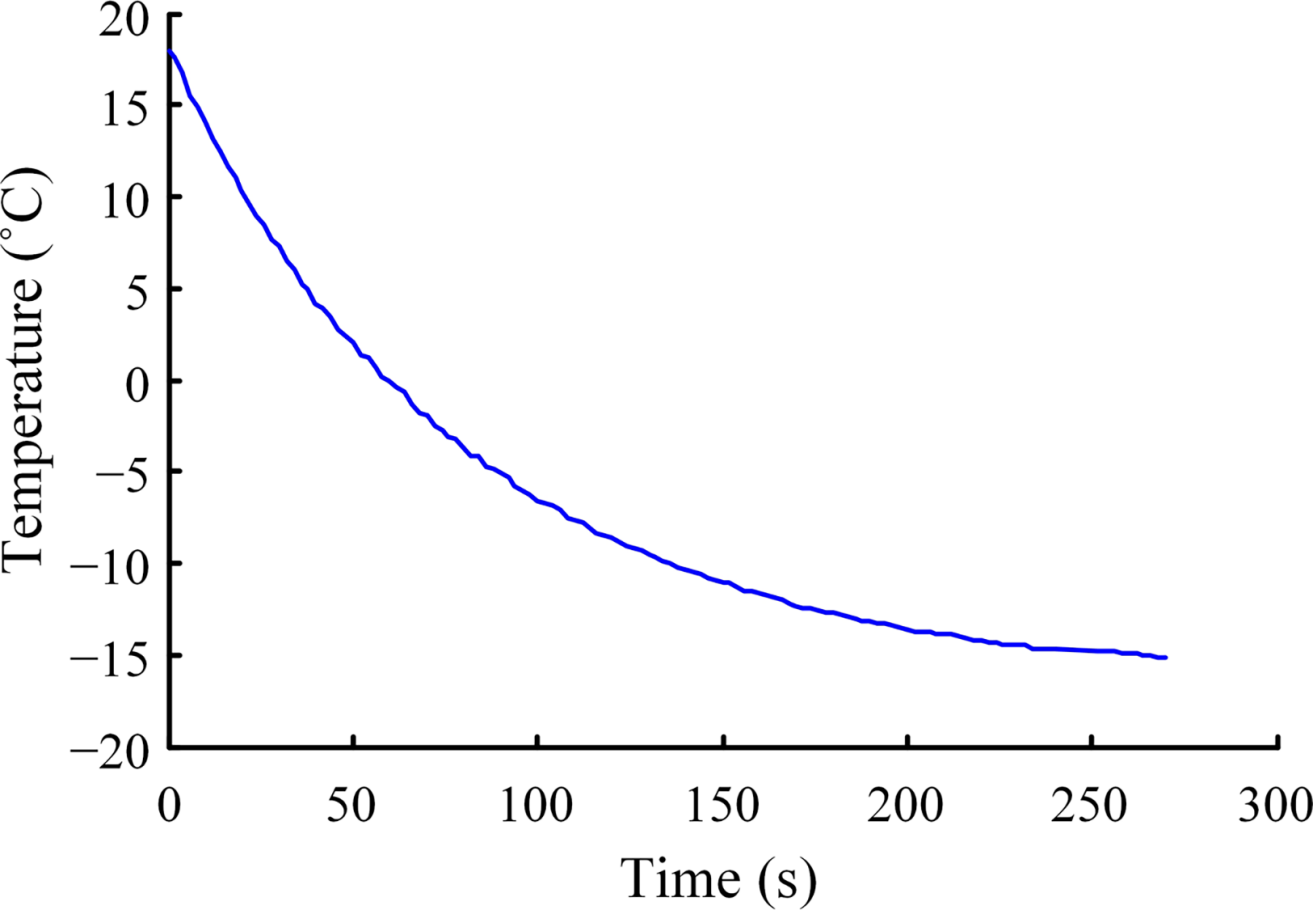
Cooling curve on substrate.

At the end of each test and before the next experiment, the substrate surface temperature was restored to ambient temperature (18°C), as measured by the K-type thermocouple located on the surface. The substrate temperature was continuously decreased during the experiment, the cooling curve is depicted in Fig 4.

## Results

### Frost formation on butterfly wing

**Fig 5:**
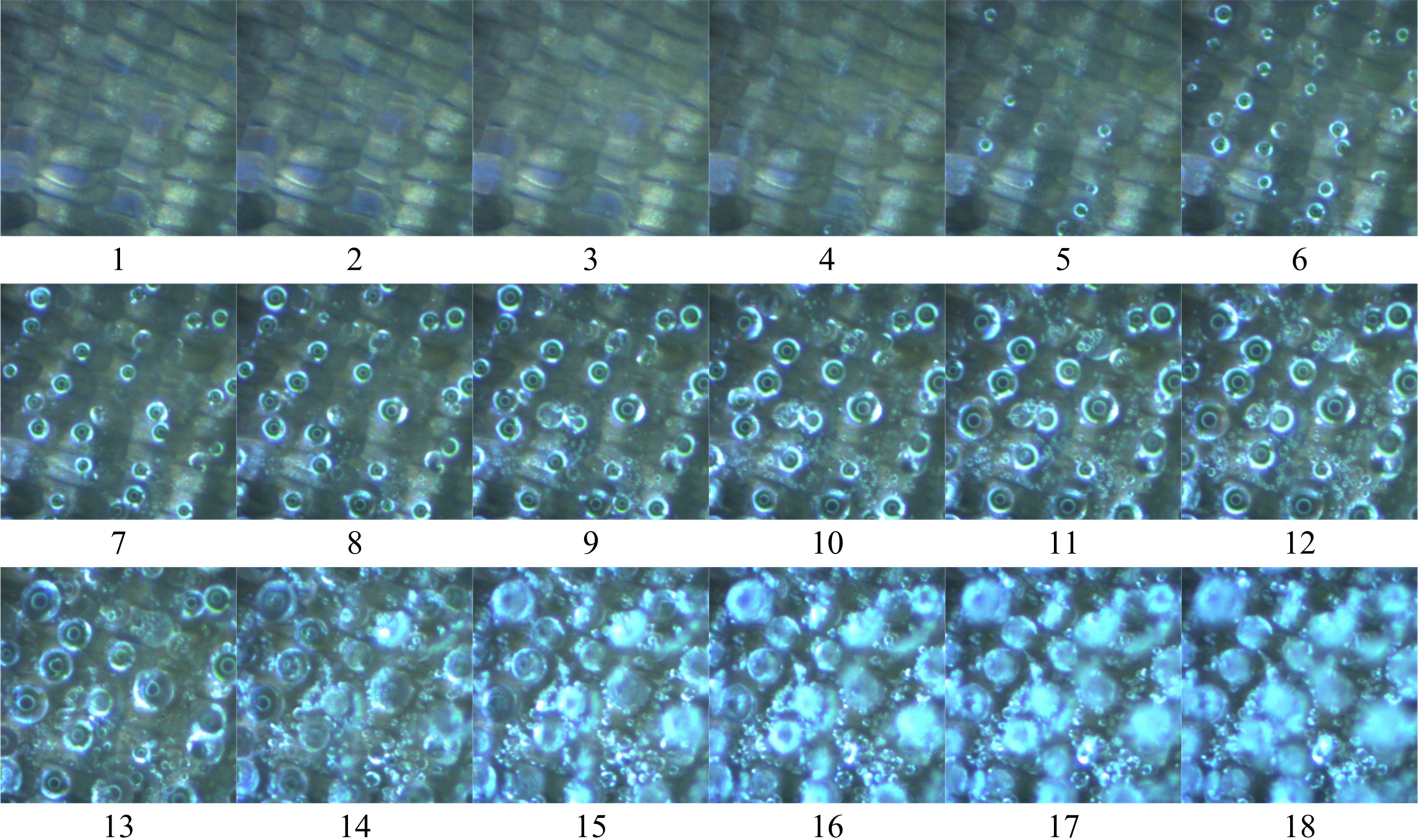
Frosting process on butterfly wing surface of *Mimathyma nycteis*.

Fig 5 shows image sequences of the frost formation process on a *Mimathyma nycteis* wing. As apparent from this figure, the early-stage of frost formation can be separated into three distinct periods, namely: the condensation, freezing, and frost growth periods.

In this experiment, the condensation period began when the surface temperature reached the dew point. Water vapour started to condense on the surface, and many micro-droplets were formed simultaneously. At the very beginning of this period, the initial micro-droplets were too small for accurate observation using the current apparatus. However, the initial condensation could be indirectly detected by observing the colour change of the surface. In the example of the frosting process on a *Mimathyma nycteis* wing shown in Fig 5 (Images 1–5), it is apparent that numerous tiny droplets gradually formed, which caused the surface to gradually turn white after cooling.

**Fig 6:**
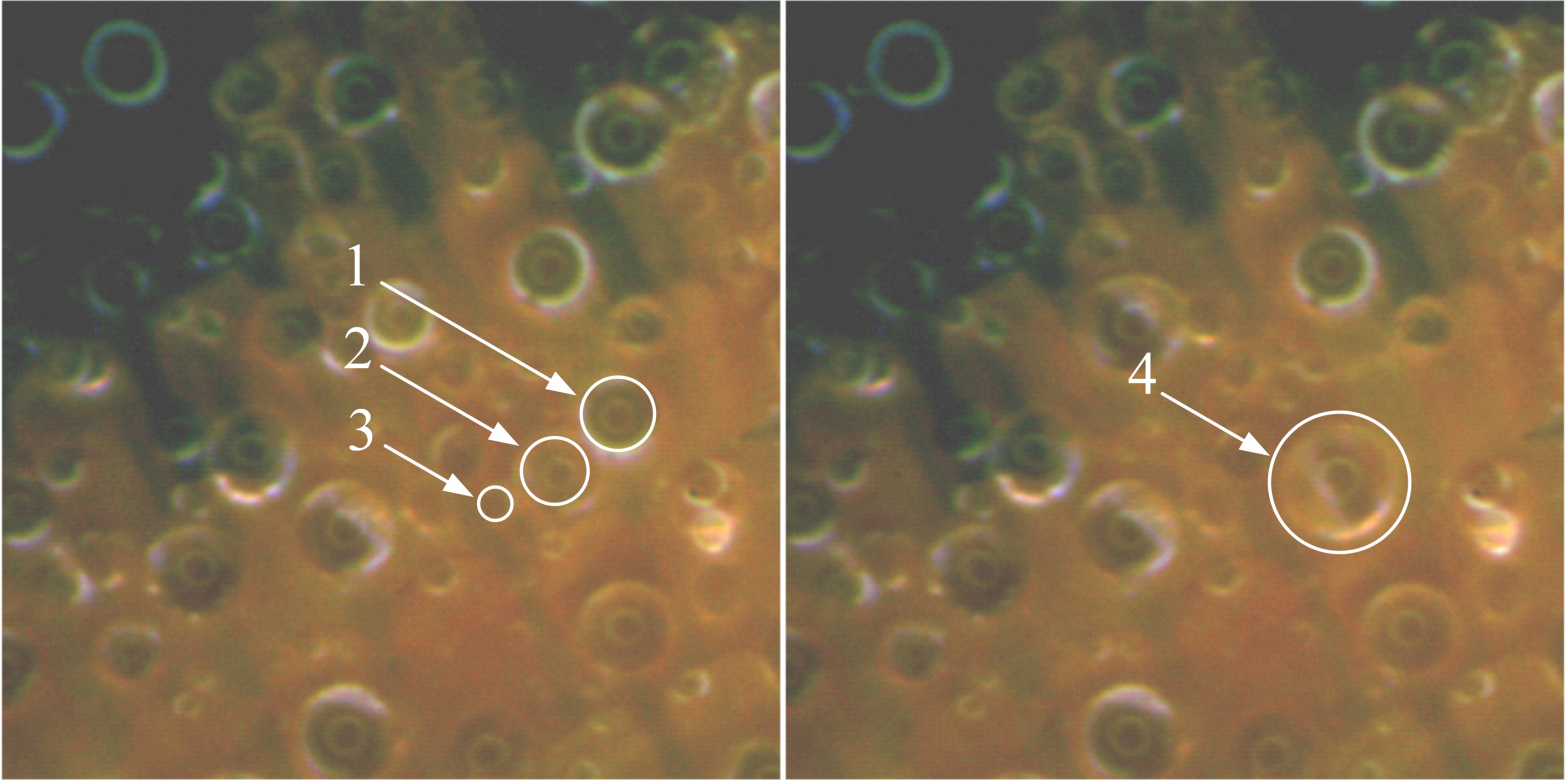
Combination of droplets on butterfly wing surface.

When the surface temperature dropped below the dew-point but remained above the freezing point, the condensation process continued; then, the droplets could grow and coalesce with nearby droplets. Two forms of coalescence occurred: emergence and combination. In the case of emergence, droplets dashed across the surface and smaller droplets merged together, this behaviour was indicated by a surface colour change (from light to dark). During this stage, two or three droplets combined at the mass center of the group. Fig 6 shows an example in which, the droplet 4 is formed by droplets 1, 2, and 3. The end of the condensation period was defined by the first appearance of a frozen droplet in the field of view, for which the transition from liquid to solid was easily visualized, with the droplet changing from transparent to milky textured. The section period of the early stage (the freezing period) was relatively short, and its end was marked by completely frozen droplets. In the last period in the early-stage of frost formation, i.e., the frost growth period, desublimation of the water vapour occurred. That is, the water vapour transformed into a solid directly.

### Durability of hydrophobicity after frosting/defrosting cycles

**Fig 7:**
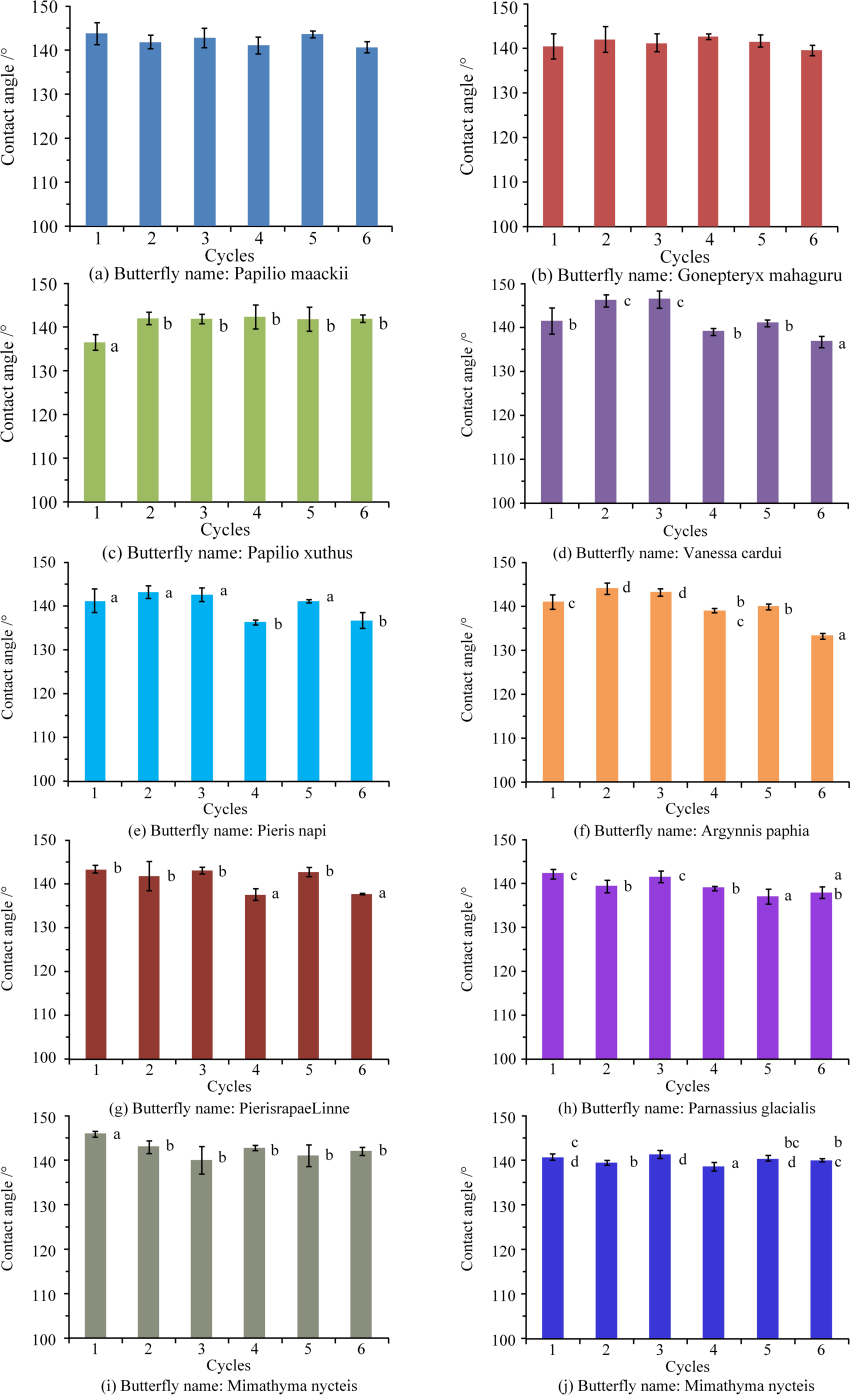
Water contact angles of butterfly wing surfaces following 0–6 frosting/defrosting cycles (Units: Degree). The mean and deviation values for each sample after each cycle are shown in the bar chart. The letters near the columns represent significant differences between the values before and after the frosting/defrosting experiments (P = 0.05). The error bars represent 95% confidence intervals.

Fig 7 shows the water contact angles of the measured butterfly wing surfaces after 0–6 frosting/defrosting cycles. Note that, generally, the contact angle measurement was imprecise, particularly as the butterfly wings surfaces have rough microstructures. Fig 7 shows that the changes in the water contact angles of the butterfly specimens following repeated frosting/defrosting processes. No significant changes are apparent, contrary to the findings of the previous studies on artificial hydrophobic surface coatings [19]. The mechanism behind this phenomenon is discussed in the following section (Discussion).

Analysis of variance (ANOVA) was used to estimate the significant difference in contact angle for each case. Note that, although the contact angles had changed, the changes were not in agreement with the number of test. Further, the measured contact angles were related to the flatness of the butterfly wing specimen which were attached to the copper sheets. In addition, the roughness of the butterfly wing surface also affects the contact angles. As a result, no significant changes in contact angle were measured in the present study.

**Fig 8:**
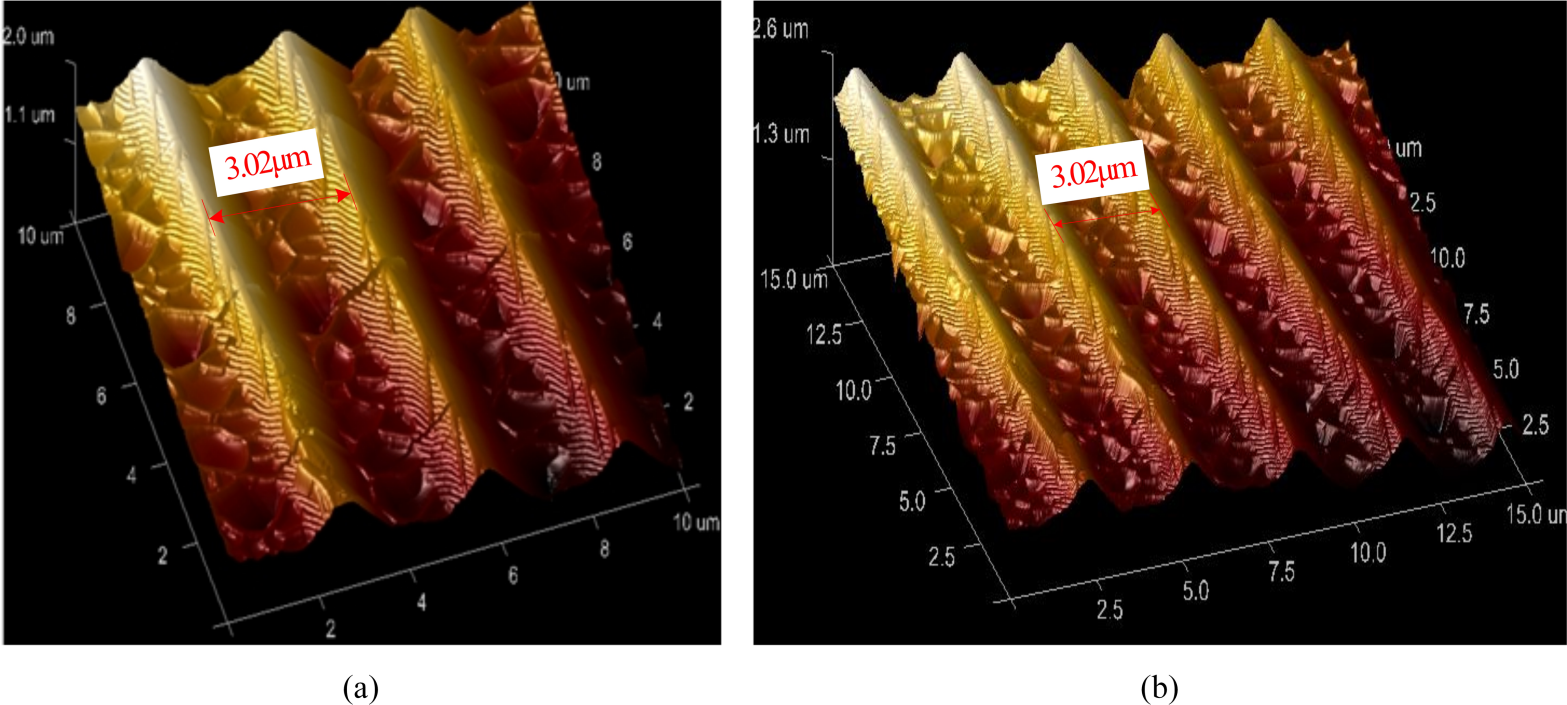
Surface morphology of *Danaus genutia* wings. (a) Before and (b) after six frosting/defrosing cycles.

After several frosting cycles, the surface structures of the specimen were characterised using a Bruker atomic force microscope. The surface morphology of the wing of the *Danaus genutia* butterfly is shown in Fig 8. Comparison of the Fig 8(A) and Fig 8(B), it reveals no obvious change in the microstructure of the wing surface. Indeed, the spacing between the longitudinal ribs remained at approximately 3.02 μm after repeated frosting/defrosting cycles. Furthermore, the microstructure was still equipped with equally spaced parallel longitudinal ribs and special transverse rib features. Thus, the repeated frost cycles did not seem to have a visible influence on the microstructure of the butterfly wing surface. In addition, the wettability of the butterfly wing did not decrease.

## Discussion

As mentioned in the Introduction, hydrophobic surfaces can restrain or alleviate frost formation. In general, the higher the water contact angle value of the surface, the better the anti-icing performance. Previously, Farhadi et al. studied the anti-icing performance of several hydrophobic coatings, found that the contact angles of the tested coatings decreased sharply with an increased number of frosting/defrosting cycles, while the coating surface gradually eroded [19]. The explanation of this degradation is that the sharp tips of the surface nanostructure on the surface are indented into the frozen liquid upon freezing. Thus, they can be damaged during frosting/defrosting, because of the interfacial stress generated during freezing. However, our analysis of butterfly wings indicates that the microstructure of the butterfly wing surface is not damaged by frosting/defrosting, and the spacing between the longitudinal ribs is not obviously changed. In our experiments, it was found that the micro-morphology penetrated the water film on the butterfly wing surface. Further, the contact angles of the butterfly specimens subjected to six frosting/defrosting cycles were measured, and no obvious the deterioration of contact angle was not obvious observed for butterfly wing surface, with minor change in contact angle. Therefore, the wettability of the butterfly wing surface was not significantly affected. Farhadi et al. have hypothesised that the super-hydrophobic nanostructure of the elastic material may improve abrasive resistance against frosting/defrosting [19]. However, we believe that there are many temperature and humidity parameters that may impact on this outcome.

**Fig 9:**
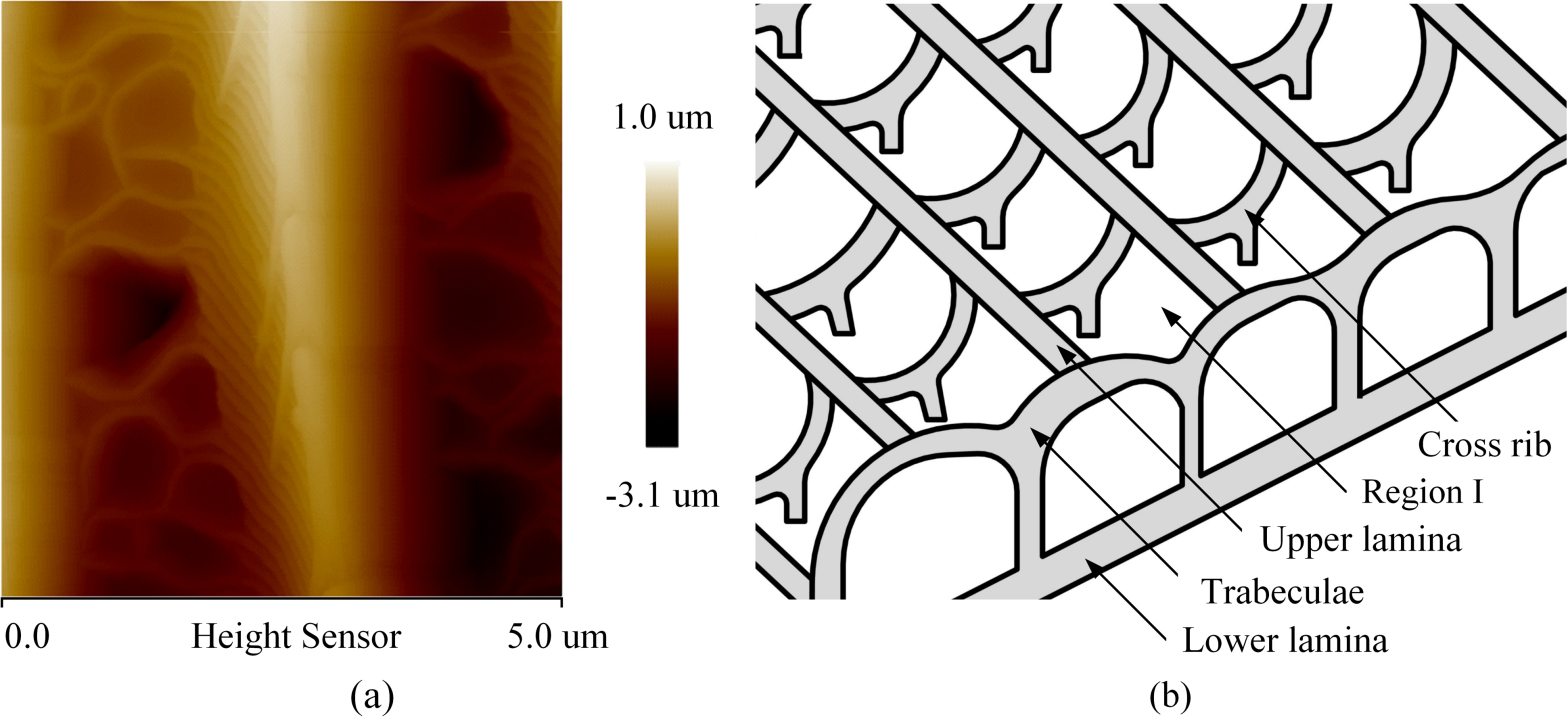
**Microstructure of butterfly:** (a) butterfly wing examined in the present experiment and (b) illustration of the observed butterfly wing structure.

Chemical composition and surface morphology are the two main factors that influence surface hydrophobicity [25–29]. However, a few published papers [16–21] indicated the hydrophobic characters of artificial surfaces decreased gradually and micro-nano structure deteriorated with increase of the freeze-thaw cycles. Thus, the present paper discusses the influence of surface structure of a butterfly wing on its hydrophobic durability. The surface morphology of a butterfly wing dominates the hydrophobic property, because the butterfly wing surface is comprised of chitin scales with an intrinsic contact angle of only 90°. Therefore, the butterfly wing surface maintains good hydrophobic characteristics because the spacing is unchanged and the microstructure on the wing surface is not damaged by the frosting/defrosting cycles, as shown in Fig 8. Panel A in Fig 9 shows the microstructure of the wing surface of the experimental butterfly; in conjunction with a previous study [30] and observed the microstructure during the test, Panel B in Fig 9 illustrates the microstructure of the butterfly wing. From Fig 9, a distinct structure with a certain spatial scale is apparent. Notably, the entire structure contains internal lacunas or hollow spaces filled with air.

In a related study, Rykaczewski and Scott [31] observed the water condensation process on nanostructures which consisted of a bunch of nano-wires. They found that the condensation in the nanostructures also exhibited a three-stage drop growth process. Firstly, a droplet with a diameter of less than 1 μ**m** condensed in the nanostructure, which was fully in Wenzel mode. Subsequently, the droplet began to emerge from the wetted area. This droplet emergence was driven by an increase in contact angle between the droplet and a base area of constant size; meanwhile a transition from Wenzel to Cassie state occurred.

Considering the characteristics in Fig 8 and Fig 9 and the phenomenon discovered by Rykaczewski and Scott [31], the following explanation for the reason for lack of significant changes in the contact angle of the butterfly wing surfaces after several frosting/defrosting cycles can be given.

(1) With a decrease in substrate temperature, the temperature of the butterfly wing structure gradually drops to the dew point, and the temperature reduction proceeds from the lower (lower lamina) to upper surface (upper lamina). The temperature reduction process is slow and take some time, because the chitin of the butterfly wing surface is a low thermal conductivity material. There are two possible scenarios here. Firstly, when the temperature of the upper lamina reaches the dew point, the droplets growing in the wing structure may be insufficiently large to fill the lacunas or hollow spaces. Subsequently, the droplets condense on the upper surface, generating a shielding effect and preventing the water vapour from continually entering the lacunas. Lacunas that are not filled with droplets reduce the thermal conductivity of the butterfly wing, and delay the temperature transfer. The second possibility is that, the droplets growing in the structure (region I, Fig 9(B)) may flood the lacunas or hollow spaces. Then, the droplets may achieve the approximately spherical shapes and emerge from the nanostructure. (2) When the temperature decreases to the freeze point, the droplets are frozen, and the ice crystals would be getting bigger and increasing the size of the droplets.

For either case mentioned above, it is difficult to destroy the spatial structure of the butterfly wing surface. The frozen droplet sizes cannot exceed the volume of the lacunas, and the elasticity of the chitin allows the wing structure to bear a certain deformation.

In summary, comparison of the artificial nanostructure surfaces investigated in previous studies [19, 31] with the topography of a typical butterfly wing surface has highlighted the special space structure of the butterfly wing. That is, the wing contains a large inner space that can easily accommodate frozen droplets without damaging the wing structure; thus, the butterfly wing surfaces still retain good hydrophobicity following repeated frosting/defrosting cycles.

## Conclusion

From formation on the surfaces of wings from different butterfly species was investigated in his study, to examine the durability of the wing hydrophobicity. Both observation and experimental results show that the early-stage of frost formation on a butterfly wing surface can be divided into three periods: the condensation, freezing, and frost growth periods. As regards to the durability of the hydrophobicity, no apparent changes were found in the water contact angles on the butterfly specimens before and after frosting/defrosting processes. The special micro-space structure of the butterfly wing surface was not damaged after six frosting/defrosting cycles. In addition, the micro-space structure decreases the thermal conductivity of the butterfly wing surface and delays the frosting time. This behavior yields improved durability of the butterfly wing hydrophobicity.

This study elucidates the frost formation process on butterfly wing and the hydrophobic durability mechanism of a butterfly wing. The findings also provides an experimental basis for the design of new and durable anti-icing surface coatings to suppress frost/ice growth, which could have industrial applications.

In addition, investigation of the frosting/icing process and hydrophobicity mechanism of a natural organism will broaden research horizons and scopes. Such analysis will provide a new direction towards enhanced durability for hydrophobic surfaces, facilitating the fabrication of the hydrophobic surfaces with good durability using bionics and nature inspired surfaces.

## Supporting information

S1 Dataset The water contact angles of the butterfly surface after subjected to frosting/defrosting cycles. (XLSX)
