# Correction: Hydrophobic durability characteristics of butterfly wing surface after freezing cycles towards the design of nature inspired anti-icing surfaces

**DOI:** 10.1371/journal.pone.0194956

**Published:** 2018-03-21

**Authors:** The PLOS ONE Staff

## Notice of Republication

An incorrect version of Fig 4 was published in error. This article was republished on February 6, 2018 to correct for this error. The publisher apologizes for the error. Please download this article again to view the correct version.
